# Antioxidant and Quality Effects of Red Grape Pomace in Barbecued Pork Burgers: Implications for PAH Formation

**DOI:** 10.3390/antiox14070832

**Published:** 2025-07-07

**Authors:** María Jesús Petrón, María Jesús Martín-Mateos, Miriam Sánchez-Ordóñez, Belén Godoy, María Rosario Ramírez-Bernabé

**Affiliations:** 1 Escuela Ingenierías Agrarias, Universidad de Extremadura, 06006 Badajoz, Spain; 2 Centro de Investigaciones Científicas y Tecnológicas de Extremadura (CICYTEX), Instituto Tecnológico Agroalimentario (INTAEX), 06006 Badajoz, Spain; mariajesus.martinmat@juntaex.es (M.J.M.-M.); miriam.sanchezo@juntaex.es (M.S.-O.); belen.godoy@juntaex.es (B.G.); mariarosario.ramirez@juntaex.es (M.R.R.-B.)

**Keywords:** grape pomace, lipid oxidation, barbecued burgers, polycyclic aromatic hydrocarbons, PAHs

## Abstract

The growing concern over the presence of polycyclic aromatic hydrocarbons (PAHs) in grilled meats has intensified the search for natural mitigation strategies. This study evaluates the effect of red grape pomace (RGP), a natural by-product with antioxidant properties, on the lipid stability, color, fatty acid profile, volatile compounds, and PAHs formation in barbecued pork burgers. Unlike previous studies focusing on polyphenol extracts, this work investigates, for the first time, the direct incorporation of whole RGP stabilized by high hydrostatic pressure (HHP), a method that preserves its bioactive profile and ensures food safety. Incorporation of RGP at different levels (0.5%, 1%, and 3%) demonstrates its potential as a functional ingredient in meat products. Our results show that RGP effectively inhibits lipid oxidation, as indicated by significantly lower malondialdehyde (MDA) levels (*p* < 0.001) compared to control batches. It also modified the fatty acid profile by reducing saturated fatty acids and increasing the linoleic acid content (up to 15.56% at the 3% level). As the RPG concentration increased, color parameters (lightness, redness, yellowness, chroma, and hue) decreased significantly (*p* < 0.001), particularly at higher pomace levels (1% and 3%). The RGP did not significantly affect the PAH concentration, indicating its safe use in barbecued products. However, it selectively influenced volatile compounds, decreasing the hydrocarbon levels at higher concentrations, likely due to its antioxidant properties. These findings suggest that stabilized RGP may serve as a natural additive that enhances the nutritional quality and reduces lipid oxidation, without promoting PAH formation in thermally processed meats.

## 1. Introduction

Polycyclic aromatic hydrocarbons (PAHs) are organic molecules primarily composed of carbon and hydrogen elements [1]. They are recognized as significant carcinogenic pollutants, posing serious health risks due to their widespread presence in the environment [2]. Human exposure to PAHs occurs through various routes, including dietary intake, inhalation, and dermal contact, making these compounds a widespread public health issue [1]. Of particular concern is the potential for long-term exposure to PAHs through the consumption of contaminated meat products, which has been linked to an elevated risk of cancer and other diseases [3]. PAHs are known to induce adverse health effects such as inflammation, immune system dysfunction, atherosclerosis, and various forms of cancer due to their ability to cross cell membranes and produce reactive intermediates with mutagenic and carcinogenic potential [1].

The formation of PAHs during food preparation is heavily influenced by cooking techniques such as smoking, grilling, barbecuing, or baking. Factors such as the type of fuel used, proximity to heat sources, cooking duration, and temperature significantly affect the PAH levels [4]. Direct cooking methods, like barbecuing, where food comes into direct contact with the heat source, lead to a higher PAH contamination, largely due to the deposition of these compounds from combustion fumes. In contrast, indirect methods, such as electric baking, result in lower PAH levels, as the food is not in direct contact with the heating [5]. However, even in indirect cooking, the pyrolysis of the food’s intrinsic components can still result in PAH formation, though to a lesser degree.

The risks posed by PAHs in food are becoming more apparent, with studies indicating that the established safety limits for PAHs are often exceeded in various food products [6]. As a result, there is an increasing need for effective strategies to reduce PAH contamination in food. Preventive measures, such as modifying food-processing techniques, reducing cooking temperatures, and avoiding direct heat exposure have been recommended to limit PAHs formation [4].

Additionally, incorporating natural antioxidants into food products has shown significant potential in reducing the formation of harmful PAHs, particularly during cooking methods involving direct heat, such as grilling and barbecuing. Antioxidants, especially those derived from natural sources like herbs, spices, and plant extracts, can effectively inhibit lipid oxidation, a critical step in PAH formation. They achieve this by neutralizing free radicals and trapping reactive intermediates, such as α-dicarbonyls, which are precursors to PAHs [7]. Natural antioxidants, such as polyphenols, flavonoids, and carotenoids, not only reduce oxidative stress but also act as protective agents by preventing the pyrolysis of fats and proteins that leads to PAH production. Studies have shown that compounds like resveratrol, quercetin, and catechins, commonly found in grape pomace, green tea, and rosemary, can significantly reduce the formation of carcinogenic PAHs when applied to meats before cooking [8,9]. These bioactive compounds help prevent the formation of harmful by-products under high-heat conditions by neutralizing reactive species and modulating heat-induced reactions, including the Maillard reaction [10].

However, most of these studies have focused on polyphenol extracts or purified compounds, whereas the use of whole food matrices such as grape pomace remains less common. Whole by-products like pomace offer additional functional benefits, such as dietary fiber, but require stabilization to ensure microbial safety and preserve bioactive compounds. High Hydrostatic Pressure (HHP) is a non-thermal food processing technology that has gained attention for its potential in the valorization of agrifood by-products. Furthermore, HHP has also been used to improve the recovery of bioactive compounds from various agro-industrial wastes. By increasing cell permeability and compound diffusivity, HHP enhances the extraction efficiency of valuable components such as phenolics [11]. Recently, the combination of HHP and blanching has been suggested as a method to stabilize red and white grape pomace, reducing microbial counts while preserving the phenol content and inactivating enzymes like polyphenoloxidase [12,13,14]. These stabilized grape pomaces have been shown to effectively delay lipid and protein oxidation in pork burgers and dry-cured sausages [15,16].

However, to our knowledge, no study has evaluated the use of whole HHP-stabilized red grape pomace (RGP) as a direct additive in barbecued meat products, nor its impact on PAH formation under grilling conditions. This represents a significant gap in the literature, especially considering the increasing demand for clean-label, multifunctional ingredients that improve both food safety and nutritional value. In the context of barbecued burgers, where the high temperatures often lead to the formation of carcinogenic polycyclic aromatic hydrocarbons (PAHs), the incorporation of stabilized grape pomace could play a crucial role in reducing these harmful compounds. The polyphenols present in RGP, such as resveratrol and anthocyanins, as noted by Antonic et al. [9], have been linked to the inhibition of lipid oxidation, which is a critical step in the development of PAHs during high-heat cooking processes. By limiting the lipid peroxidation, RGP could effectively reduce the precursors required for PAH formation.

Given the carcinogenic nature of PAHs, strengthening regulatory frameworks and establishing stricter exposure limits are essential for reducing human exposure [6]. Furthermore, research into culinary practices or ingredients that mitigate PAH formation is critical to improving public health outcomes [4].

This study aims to address this gap by testing the hypothesis that the direct incorporation of HHP-stabilized RGP into pork burgers can reduce lipid oxidation and influence PAH-related pathways during barbecuing, while also improving quality attributes such as color and fatty acid composition. The potential of RGP, including its antioxidant and nutritional contributions, makes it a promising candidate for developing safer, more sustainable meat products. The novelty of this work lies in the use of whole, processed red grape pomace as a clean-label functional ingredient within a realistic cooking context—offering a practical solution for both industry and consumers.

## 2. Materials and Methods

### 2.1. Manufacture of the Ingredient from Red Grape Pomace (RGP)

Red grape pomace (cv. Tempranillo, September 2022) was supplied by a winery in Santa Marta de los Barros (Badajoz, Spain) following a traditional red wine production process. The grapes were first pressed, and the resulting must, with skins and seeds, was fermented without the use of added enzymes. After fermentation, about 5 kg were collected as the “initial” pomace. The pomace was thermally blanched (103 °C, 1 min) using steam-based equipment with variable-speed stainless steel mesh belts to achieve the total deactivation of polyphenol oxidase (PPO); the blanching time was optimized based on the enzyme activity and phenolic content.

The blanched pomace was vacuum-sealed, frozen (−18 °C, 24 h), milled into fine powder (RETSCH ZM200, Germany), vacuum-packed in low-oxygen-permeability Eurobag plastic (polyamide/polyethylene, 20/100), and treated with high hydrostatic pressure (600 MPa, 5 min, 16 °C) using semi-industrial equipment (6000/55, Hiperbaric, Spain).

The valorized ingredient (RGP), processed via thermal blanching, milling, and HHP, was vacuum-sealed and stored at −80 °C until used in barbecued pork burgers. For the analysis, the frozen RGP was ground using a Thermomix (Vorwerk, Germany) at maximum speed for 1–2 min to ensure homogeneity.

### 2.2. Manufacture, and Cooking of Burgers

Burgers were prepared with ground pork (local market), salt (13 g/kg), garlic (1.5 g/kg), onion (1.5 g/kg), and black pepper (0.5 g/kg). Negative control (NC) burgers excluded additives; commercial formulation (CF) burgers included 0.45 g/kg sodium metabisulphite.

The red grape pomace (RGP) was added to NC at 0.5%, 1%, and 3% (*w*/*w*), based on sensory trials identifying 3% as the maximum acceptable level. The sensory trial was carried out by a trained panel of eight assessors from the Instituto Tecnológico Agroalimentario (INTAEX). Panelists evaluated six sensory attributes: general appearance, odor, flavor, texture, off-flavor, and overall acceptability. Each attribute was rated on a structured 0–10 scale (0 = low intensity, 10 = high intensity).

Five formulations were prepared (NC, CF, 0.5%, 1%, and 3% RGP), yielding 50 burgers (n = 5 per batch × 5 formulations × 2 preparations). Raw burgers had 67.4 ± 1.1% moisture, 19.5 ± 0.4% protein, and 9.0 ± 0.7% fat.

Burgers were thawed (24 h, 5 °C) and barbecued on Weber charcoal briquettes (4.82% moisture, 25.4% volatile matter, 9.3% ash, 65.3% fixed carbon, and a gross calorific value of 6425 cal/g), characterized per standards (EX)UNE-EN_1860-2, 2024 [17] and (EX)UNE-EN_ISO_18125, 2018 [18]). Briquettes were preheated (20 min, >300 °C). Cooking was performed in five batches (five burgers per formulation per batch) on a 0.22 m^2^ grill placed 8–10 cm above the briquettes. The burgers were rotated to compensate for temperature differences (center vs. edges) and cooked for 15 min (7.5 min per side) until the internal temperatures reached 98–101 °C.

Of the 50 cooked burgers, 25 were analyzed for color, moisture, and TBARS in the frozen samples; the remaining 25 were lyophilized for the volatile compound and PAH analysis.

### 2.3. Physicochemical Composition of RGP and Burgers

The proximate composition of RGP and burgers was determined using established methods. The moisture and protein were assessed using AOAC procedures [19], the fat was assessed using the Folch extraction method [20], and the fiber was assessed via the Southgate method [21]. The pH was measured with a calibrated pH meter (Hanna Instruments, Woonsocket, RI, USA), and measuring the water activity (aw) at 25 °C was performed with a Novasina Labmaster device (Lachen, Switzerland). Fatty acid methyl esters (FAMEs) from the extracted fat were analyzed using a gas chromatograph (Agilent 6890, Agilent Technologies, Santa Clara, CA, USA) with a flame ionization detector (FID), and the results were expressed as percentages of total FAMEs.

The total antioxidant activity of RGP was measured using triplicate extraction: 1 g pomace was mixed with 50 mL of MilliQ water/methanol/citric acid (80:19.9:0.1), incubated at 60 °C for 60 min, centrifuged (10,000 rpm, 4 °C, 15 min), evaporated (20 min), and adjusted to 50 mL with MilliQ water. An aliquot (50 µL) was mixed with 1 mL of ABTS reagent (Sigma-Aldrich, Madrid, Spain), and the absorbance was measured at 750 nm (UV-2401 PC Shimadzu spectrophotometer, Shimadzu Scientific Instruments, Kyoto, Japan). Results were calculated against a Trolox standard curve (0–350 µM) and expressed as mmol Trolox g^−1^.

The total phenolic content of the RGP was determined with a Folin–Ciocalteu colorimetric assay [22], using a gallic acid standard curve (Merck, Madrid, Spain) from 0 to 16 ppm, and expressed as mg GAE 100 g^−1^ (wet base).

### 2.4. Analysis of Barbecued Pork Burgers

Instrumental color: The CIELAB instrumental color determinations were performed using a Minolta CM-5 spectrophotometer (Minolta Camera, Osaka, Japan), with an illuminant/angle of D65/100 and a measuring area of 30 mm. The color coordinates (lightness (L*), redness (a* red/green axis), and yellowness (b* yellow/blue axis)) in the CIE Lab color space were analyzed. In addition, the hue angle was calculated (h° = tan − 1·(b*/a*)) as well as the saturation index or chroma (C*) (C = (a*·2 + b*·2)·0.5). A total of four color measurements were taken for each burger (two on each side), and the final value was determined as the mean of the readings.

Oxidative stability: Lipid oxidation was measured using the thiobarbituric acid reactive substances (TBA-RS) method as described by Sorensen & Jorgensen [23]. TBARS values were based on a standard curve prepared with 1,1,3,3-Tetraethoxypropane (TEP), and the results were reported as milligrams of malondialdehyde per kilogram of the sample (mg MDA kg^−1^).

Volatile compounds: Two grams of the ground, freeze-dried, cooked burger were placed into a 20 mL screw-capped vial with a Teflon-silicone septum. Volatiles were extracted from the headspace using a 1 cm 50/30 μm DVB/CAR/PDMS SPME fiber (Supelco, Bellefonte, PA, USA) at 37 °C for 30 min. Desorption was carried out in the GC injection port at 270 °C for 600 s in splitless mode.

Compounds were separated on a Varian CP-3800 gas chromatograph with a CombiPAL autosampler (CTC Analytics, Zwingen, Switzerland) and a HP-5 capillary column (30 m × 0.32 mm × 0.25 μm; Agilent Technology, Santa Clara, CA, USA). The injection port temperature was 270 °C. The oven program: 35 °C (10 min), ramped to 250 °C at 7 °C/min, held for 5 min (total 45 min). Transfer line, trap, and manifold temperatures were 280 °C, 200 °C, and 60 °C, respectively. Detection was performed using a Varian Saturn 2200 MS (Varian Inc., Palo Alto, CA, USA) with electron impact ionization (70 eV), scanning 40–300 m/z at one scan/sec.

Volatiles were identified by mass spectra and linear retention indices (LRI) compared to commercial standards (Sigma-Aldrich, St. Louis, MO, USA) or the NIST library (Agilent MSD Chemstation E.02.01.1177). Quantification used 4-methyl-1-pentanol as internal standard (1.22 mg kg^−1^), with results expressed as μg kg^−1^.

Policyclic aromatic hydrocarbons. PAHs were analyzed following Onopiuk et al. [24]. Freeze-dried minced samples (2 g) were weighed and extracted with 20 mL n-hexane in Falcon tubes, sonicated for 1 h at room temperature, filtered into round-bottom flasks, and evaporated to near dryness. Residues were dissolved in 3 mL n-hexane.

Purification was conducted using Mega BE-SI silica columns (5 g, 12,256,026, Agilent Technologies), preconditioned with n-hexane and dichloromethane. The 3 mL sample was applied, followed by 8 mL of 70:30 n-hexane (discarded), and 9 mL of a PAH-containing fraction (collected). This fraction was evaporated under nitrogen, reconstituted in 1.3 mL acetonitrile, filtered (0.45 µm), and transferred to the vials.

For quantification, a PAH calibration mix (CRM47940) containing 4–40 ppb of Naphthalene, Acenaphthylene, Acenaphthene, Fluorene, Phenanthrene, Anthracene, Fluoranthene, Pyrene, Benz[a]anthracene, Chrysene, Benzo[b]fluoranthene, Benzo[k]fluoranthene, Benzo[a]pyrene, Indeno[1,2,3-cd]pyrene, Dibenz[a,h]anthracene, and Benzo[ghi]perylene was added to duplicate samples.

Separation and detection were performed on an Agilent Technologies 1100 HPLC-DAD-FLD system with a Gemini NW-C18 column (Phenomenex, 150 mm × 4.6 mm, 3 μm, 1 mL/min). Mobile phases were water (A) and acetonitrile (B). Phase B increased from 60% to 100% over 30 min, then decreased to 40% at 45.5 min. The column temperature was 30 °C; injection volume, 10 µL. Fluorescence detection used a 260 nm excitation and emissions at 330, 350, 440, and 500 nm.

### 2.5. Statistical Analysis

Five independent samples were evaluated per batch. A one-way ANOVA was used to assess the effect of treatment (NC, CF, 0.5% RGP, 1% RGP, 3% RGP) on the measured parameters, with treatment as a fixed effect. When significant differences were found (*p* < 0.05), a Tukey’s post hoc test was applied for pairwise comparisons. Analyses were performed using SPSS version 21.0 (SPSS Inc., Chicago, IL, USA).

## 3. Results

### 3.1. Characterization of the Valorized Red Grape Pomace (RGP)

The proximate composition of the RGP ingredient is shown in Table 1, including moisture (57.0%), fiber (25.4%), fat (3.9%), and protein (4.3%). The pH was lower than 4.0, and the water activity (aw) was 0.980.

The fatty acid profile of RGP showed linoleic acid (C18:2 ω6, 64.5%) as the predominant fatty acid, followed by oleic acid (C18:1 ω9, 19.4%), palmitic acid (C16:0, 9.5%), stearic acid (C18:0, 4.6%), and linolenic acid (C18:3, 1.0%).

Table 1 also reports an antioxidant activity of 45.3 mM Trolox/mL and a total phenolic content of 486.0 mg/100 g.

### 3.2. Effect of the Incorporation of the RGP in Burgers

#### 3.2.1. Composition and Fatty Acid Profile

The burgers were analyzed for moisture, protein, fat content, and fatty acid profiles (Table 2). The moisture content ranged from 55.0% to 57.8% across formulations, with no significant differences (*p* = 0.273). The protein content ranged from 29.7% to 32.2%, also without significant differences (*p* = 0.346). The fat content showed significant variation (*p* = 0.005), with the 1% and 3% RGP batches containing higher fat levels (6.9% and 6.8%, respectively) compared to the NC and CF (5.7% and 5.5%, respectively). The 0.5% RGP batch had an intermediate fat content (6.1%).

Significant differences in fatty acid composition were observed across burger batches, particularly in the 3% RGP group. The inclusion of RGP reduced saturated fatty acids, with the 3% RGP batch showing lower palmitic acid (C16:0) and arachidic acid (C20:0) levels compared to the NC and CF (*p* = 0.001). Linoleic acid levels increased with RGP addition, reaching 15.56% in the 3% RGP batch. Despite RGP’s high oleic acid content, the 3% RGP batch displayed a slight reduction in oleic acid (43.83%) compared to the NC and CF (*p* < 0.001).

#### 3.2.2. Color and Lipid Oxidation

The inclusion of red grape pomace (RGP) at different levels affected both instrumental color and lipid oxidation in barbecued burgers (Table 3).

Significant changes in all color parameters (L*, a*, b*, chroma, and hue) were observed among treatments (*p* < 0.001). As the RGP concentration increased, the lightness, redness, yellowness, chroma, and hue values decreased. The commercial formulation (CF, with sodium metabisulfite) exhibited the highest a* value, indicating greater redness. The lightness (L*) decreased with a higher RGP inclusion, reaching 46.0 for the 1% RGP and 46.4 for the 3% RGP, indicating a darker appearance. The yellowness (b*) also decreased, with the 3% RGP batch showing the lowest value (9.4). The redness (a*) declined from 7.9 in the CF batch to 4.2 in the 3% RGP batch.

Lipid oxidation, measured by the malondialdehyde (MDA) content, differed significantly among treatments (*p* < 0.001). The NC and CF groups had higher MDA values (0.4 and 0.4, respectively), whereas pomace-treated batches (0.5%, 1%, and 3% RGP) showed reduced lipid oxidation, with MDA values as low as 0.1 in the 0.5% and 1% RGP batches.

#### 3.2.3. Polycyclic Aromatic Hydrocarbons (PAHs)

The analysis of polycyclic aromatic hydrocarbons (PAHs) in barbecued RGP burgers detected only two compounds (phenanthrene and fluorene) across all batches, including the NC, CF, and burgers with the three levels of RGP (Table 4). To validate the effectiveness of the analytical method, selected samples were spiked with low levels of standard PAHs, and all target compounds were successfully detected using certified reference materials. The method showed high sensitivity, with LODs of 0.1056 µg kg^−1^ and 0.0528 µg kg^−1^, and LOQs of 0.3199 µg kg^−1^ and 0.1601 µg kg^−1^ for fluorene and phenanthrene, respectively. This confirms that the absence of other PAHs was not due to methodological limitations but rather to their actual absence in the samples under the specific cooking conditions applied. This absence is consistent with the relatively low fat content of the burger matrix and the controlled grilling method employed, both of which are known to limit PAH generation.

Phenanthrene levels ranged from 0.4 to 0.9 µg kg^−1^, while fluorene levels remained constant at 0.1 µg kg^−1^ across all samples, indicating a low PAH production in the barbecued pork burgers.

No significant differences in the PAH levels were observed among treatments (*p* = 0.173 for phenanthrene; *p* = 0.605 for fluorene).

#### 3.2.4. Volatile Compounds

The inclusion of red grape pomace (RGP) in barbecued burgers caused significant changes in the volatile compound profiles compared to control groups. Out of 70 volatile compounds identified, 32 showed significant differences among treatments (Table 5). Volatile compounds were grouped into sulfur compounds, terpenes, alcohols, aldehydes, ketones, linear and aromatic hydrocarbons, acids, and others.

Aldehydes: Hexanal was the most abundant aldehyde identified. The negative control exhibited the highest hexanal concentration (2809.5 µg kg^−1^), significantly higher than the other groups (*p* = 0.003). Burgers containing RGP showed lower hexanal levels compared to the NC group, indicating reduced lipid oxidation. In contrast, 2-Decenal (Z) concentrations increased significantly with pomace addition, particularly in the 1% and 3% RGP groups (34.8 and 32.2 µg kg^−1^, respectively; *p* = 0.015).

Hydrocarbons: Significant differences in hydrocarbons were observed across treatments. Hexane, 3-methyl was elevated in the 0.5% RGP group (65.5 µg kg^−1^; *p* = 0.008), while cyclohexane, methyl- peaked at 1% RGP (70.6 µg kg^−1^; *p* = 0.039) and hexane, 2,4-dimethyl at 0.5% RGP (201.7 µg kg^−1^; *p* = 0.018). Similarly, 3-ethylhexane peaked in the 0.5% RGP group (72.4 µg kg^−1^; *p* = 0.004), and 1-Octene, 6-methyl was present in the 0.5% RGP group but absent in the 3% RGP samples (*p* < 0.001).

Ketones: The ketones 2-Pentanone, 3-methyl- and 2-Hexanone, 4-methyl- were found at higher levels in the negative control and lower RGP groups. Their concentrations significantly decreased in the 3% RGP burgers.

Alcohols: The alcohol 1,3-Butanediol showed a significant increase at the 0.5% RGP level (29.5 µg/kg; *p* < 0.001), followed by stabilization or reduction at higher RGP concentrations (1% and 3%).

Sulfur and Nitrogen Compounds: Sulfur compounds, such as 1-Propene, 3,3′-thiobis-, increased with the 0.5% RGP addition. In contrast, certain nitrogen compounds, like pyrazines, were absent at higher RGP concentrations, indicating a possible suppression of heat-induced nitrogen volatiles at higher pomace levels.

Terpenes: Only four terpenes exhibited significant changes with the RGP addition. 3-Thujene, β-Myrcene, 3-Carene, and Copaene levels increased in burgers with RGP or metabisulfite (CF), suggesting some preservation of terpene compounds in the presence of antioxidants.

## 4. Discussion

### 4.1. Characterization of the Valorised Red Grape Pomace (RGP)

The low pH (<4.0) and high Aw (0.980) suggest potential long-term stability for RGP, with previous studies indicating a shelf-life of at least nine months under similar conditions [12]. The low pH also contributes to stabilize anthocyanins, further preserving the ingredient’s quality [25].

The high fiber content aligns with findings from Ramírez et al. [14] for white grape pomace (27.8%), while the moisture content is comparable to other red grape pomaces, such as Petit Verdot and Tempranillo cultivars [12]. The protein content in this RGP was slightly higher than in other valorized red grape pomace samples (3.4 [13]).

Variability in the pomace composition is common, largely due to factors like grape maturity at harvest and winemaking processes, as noted in studies by García-Lomillo & González-San José [26]. All values here fall within ranges reported for similar ingredients [9,12,13]. However, comparisons are challenging, as compositions are often given on a dry basis or derived from the flour of grape pomace [8,27,28], and sometimes, only skins are used without seeds or stalk remains [29,30].

The fatty acid profile, dominated by unsaturated fatty acids such as linoleic and oleic acids, is consistent with previous reports for grape pomace [31,32,33]. The high levels of unsaturated fatty acids, particularly linoleic acid and oleic acid, along with the presence of omega-3 fatty acids and a low saturated fat content, underscore RGP’s potential as a beneficial food ingredient linked to anti-inflammatory and cardiovascular health benefits [34].

The antioxidant activity (45.3 mM Trolox/mL) and total phenolic content (486.0 mg 100 g^−1^) are in line with values reported in the literature, confirming the rich polyphenolic composition and antioxidant capacity of grape pomace [32,33]. These authors highlight the presence of phenolic compounds, such as flavonoids, phenolic acids, and tannins, which are known to scavenge free radicals and mitigate oxidative stress.

Overall, these results justify the investigation of RGP as a key ingredient not only for its nutritional value but also for its potential role in modulating oxidative and heat-induced reactions in meat systems, a hypothesis explored in subsequent sections of this study.

### 4.2. Effect of the Incorporation of the RGP in Burgers

#### 4.2.1. Composition and Fatty Acid Profile

Despite the high moisture content of the RGP (57.04%, Table 1), its up-to-3% inclusion did not affect the final burger moisture, helping to maintain the desired texture and juiciness, which are critical for consumer satisfaction of barbecued products [35].

Similarly, the protein content remained stable despite the low protein concentration of the RGP (4.3%, Table 1), supporting the formulation’s structural and sensory properties.

A higher fat content in 1% and 3% RGP batches suggests that fiber and polyphenols from RGP improved fat retention and reduced cooking losses, increasing the juiciness and texture [36]. Polyphenols stabilize proteins, enhancing lipid retention during cooking [37,38,39]. Similar effects were observed with fiber-rich ingredients like oat and citrus, which enhance hydration and fat retention [40,41,42].

The fatty acids profile shown in Table 2 is consistent with the profiles reported in other studies on pork burgers [43]. The reduction in saturated fatty acids and increase in linoleic acid content with the RGP addition aligns with the high polyunsaturated fatty acid (PUFA) profile of the RGP (Table 1).

The slight decrease in oleic acid despite the RGP’s richness may reflect a redistribution favoring linoleic acid, altering the overall fatty acid balance. This agrees with previous findings where fruit-based additives, such as cherry, modified burger fatty acid profiles [44], highlighting the RGP’s role in enhancing the PUFA content. Our results support the working hypothesis that the RGP contributes to an improved lipid profile, which may influence the generation of lipid-derived reaction products during cooking.

#### 4.2.2. Color and Lipid Oxidation

Sodium metabisulfite (SM) effectively preserved meat redness by stabilizing myoglobin, as seen in the highest a* value in the CF batch. This effect aligns with studies on SM’s antioxidant role in protecting color in meat products, as demonstrated in research on pork burgers by Pfukwa et al. [45] and Beya et al. [46].

The darker appearance observed with the higher RGP inclusion is likely due to the presence of natural pigments such as anthocyanins. This effect has also been noted in meat products supplemented with cherry and grape extracts [44] and pomegranate rind powder [47]. Although RGP darkens the burgers, it reflects the natural coloring properties of polyphenol-rich ingredients.

The reduction in b* values (yellowness), along with decreased chroma and hue, parallels findings with other plant-based antioxidants like rosemary and tea catechins, which also altered the meat color [9,48].

The decline in redness (a*) with the RGP addition suggests that, unlike SM, natural antioxidants introduce darker pigments without maintaining the same degree of redness. Similarly, Mitsumoto et al. [49] found that tea catechins, a natural antioxidant, led to a reduction in redness in chicken meat patties, indicating that natural antioxidants, while preserving quality, may also alter color due to their inherent pigmentation. These findings suggest that, although antioxidants slow oxidation and maintain meat quality, they can introduce darker pigments from plant extracts, thereby altering the appearance.

Regarding lipid oxidation, the RGP effectively reduced MDA levels, indicating its antioxidant capacity. This reduction in MDA levels demonstrates the antioxidant efficacy of the RGP, aligning with previous findings in barbecued- and grilled-meat studies. Similar reductions in the lipid oxidation have been reported with natural antioxidant inclusions, such as rosemary and ginger powder as antioxidants in cooked pork burgers [43,50]. These antioxidants prevent oxidative degradation and maintain the nutritional quality of grilled meat products by significantly lowering the MDA levels [51]. These results provide evidence that natural antioxidants like RGP can significantly mitigate oxidative processes in high-heat cooking conditions.

In contrast, sodium metabisulfite (SM) exhibited no significant effect on the lipid oxidation in our study. This observation is consistent with its classification as a secondary antioxidant, which may act as a reducing agent or metal chelator. However, its efficacy in lipid systems is often limited under complex conditions, such as high-temperature cooking [52,53]. This reinforces the superior antioxidant functionality of RGP in the context of lipid stability.

#### 4.2.3. Polycyclic Aromatic Hydrocarbons (PAHs)

According to Commission Regulation (EC No. 1881/2006), benzo(a)pyrene and PAH4 compounds are the primary markers for PAH occurrence in food. Since neither phenanthrene nor fluorene are included in PAH4, their presence does not affect the compliance with food safety standards [54].

Phenanthrene and fluorene are considered light PAHs, which are PAHs with fewer aromatic rings (typically 2–3) and lower molecular weights. These lighter PAHs are more volatile and generally form more readily under high-heat conditions, such as grilling, compared to heavier PAHs with more rings (four or more), which are more stable and persist longer in the environment. Light PAHs are also found in higher concentrations across different meat types than heavier PAHs, likely due to the quick pyrolysis of fats during intense cooking methods [55].

The absence of significant differences in PAH concentrations among treatments suggests that the RGP addition did not influence the PAH formation. This finding is important, as it indicates that the RGP reduced the lipid oxidation without increasing harmful PAH levels, thus aligning with the food safety regulations and improving the nutritional quality.

While the addition of RGP at different concentrations (0.5%, 1%, 3%) did not significantly alter the PAH levels, this lack of variation was also observed between control samples without RGP. Therefore, the absence of PAH differences does not appear to reflect a threshold or non-linear polyphenol effect but rather the overall low PAH formation under the selected cooking conditions. This suggests that the experimental setup itself, characterized by a moderate grilling temperature and low fat matrix, was not conducive to PAH generation, making it difficult to observe any inhibitory effect attributable to RGP.

Comparing our results with similar studies is difficult, as the formation of PAHs in cooked meat can be influenced by various factors, including animal meat, cooking methods, temperature, presence of fat, or antioxidative activities [6,7,55,56].

In a previous study on roasted pork, seven PAHs were detected at a total concentration of 7.4 µg kg^−1^. The addition of dried fruits (200g per kg of pork), such as cranberries, apricots, and prunes, showed a marked inhibitory effect on PAH levels, reducing the total PAH content by 58%, 35%, and 48%, respectively. This reduction was attributed to the high polyphenol content in the dried fruits, which effectively scavenged free radicals that contribute to PAH formation during roasting [57]. However, the percentage of added ingredient in that study was much higher than the amount of RGP included in our burger formulation.

Light PAHs have been shown to be significantly influenced by the fatty acid profile [55]. In our study, the addition of the RGP ingredient to burgers significantly altered the fatty acid profile (Table 2), increasing the proportion of unsaturated fats. However, these changes did not match with differences in the PAHs formation during cooking, as the levels of light PAHs, such as phenanthrene and fluorene, remained consistent across treatments. The relationship between fatty acids and PAH formation is complex; both the fat content and the fat composition can affect the PAH levels in cooked meats [55]. These authors reported that unsaturated fats generally produce fewer PAHs than saturated fats under grilling conditions. Similarly, Kazerouni et al. [56] suggested that reducing the fat content in meat products could help low PAH formation.

In the above-mentioned studies, the authors generally report higher levels than in our study. The low levels of PAHs in this study could be attributed to the different compositions of the burger (low fat content of burger, utilization of spices with antioxidant activity in the formulation, which reduces PAHs formation) and the cooking method/temperature used (relatively low temperature), with these parameters having a strong influence on the presence of PAHs [2].

In our study, while the RGP addition slightly increased the overall fat content, the change in the fatty acid composition towards more unsaturated fats might have counterbalanced the potential for increased PAH formation, providing some oxidative stability. Furthermore, the antioxidant properties of the RGP ingredient, which significantly reduced lipid oxidation, also played a role in mitigating the PAH formation. This is supported by different authors, who observed that the inclusion of different natural plant extracts did not increase or even reduce the PAH levels in meat products [24,57,58,59].

These protective effects may be explained by the known biochemical and thermodynamic mechanisms through which polyphenols inhibit PAH formation. Polyphenols are known to exert their inhibitory effect on PAH formation through multiple biochemical and thermodynamic mechanisms. Primarily, they act as free radical scavengers, neutralizing reactive oxygen species and lipid radicals that serve as precursors for PAHs during high-temperature cooking [52]. Additionally, polyphenols can chelate transition metal ions (e.g., Fe^2+^, Cu^2+^), which catalyze Fenton-type reactions, thereby limiting oxidative degradation and radical formation in lipids [60]. Thermodynamically, polyphenols may also stabilize unsaturated fatty acids by lowering the activation energy for lipid peroxidation, reducing the extent of thermal decomposition that contributes to PAH precursors [61]. Furthermore, in complex food matrices like meat, polyphenols can interact with proteins and Maillard reaction intermediates, modulating heat-induced pathways that are linked to PAH formation [62]. These mechanisms jointly explain the protective effects observed in RGP-enriched burgers, even under grilling conditions.

In addition to these mechanisms, recent studies highlight that several volatile compounds arising from lipid oxidation, such as hydroperoxides, aldehydes, and short-chain hydrocarbons, serve as key intermediates in PAH formation during high-temperature cooking. These volatiles are generated during early thermal degradation stages and may undergo cyclization and condensation reactions, contributing to the PAH synthesis in meat matrices [63]. In line with this, Beriain et al. [64] reported that reducing the generation of volatile organic compounds like aldehydes and hydrocarbons, whether through antioxidant strategies or alternative grilling techniques, significantly lowered the PAH concentrations in cooked meats. These findings support the idea that antioxidant-mediated modulation of volatiles may indirectly suppress PAH formation through inhibition of precursor availability [64].

Such protective effects are likely driven by the combined action of polyphenols, which limit radical formation and stabilize lipids, as well as by the modulation of volatile precursors involved in PAH synthesis. This dual mechanism may explain the favorable outcome observed in RGP-enriched burgers, even under conventional charcoal grilling conditions, where the risk of PAH formation is typically higher due to pyrolysis and fat dripping on embers [63,64].

#### 4.2.4. Volatile Compounds

The volatile compounds identified are consistent with those typically found in cooked meats [65,66,67].

The reduction in hexanal levels in RGP burgers is consistent with findings from other studies where natural antioxidants have been shown to inhibit the formation of hexanal in cooked pork patties [50]. However, the increase in specific aldehydes, like 2-Decenal (Z), in burgers with a high RGP content suggests that while the overall lipid oxidation is reduced, certain secondary oxidation products may still be generated, though at lower levels. This selective inhibition pattern is consistent with observations by Muchtaridi et al. [68], who noted that natural antioxidants can selectively inhibit certain oxidative pathways while allowing others to continue.

The hexanal content correlated with TBA-RS values in burgers (which measure the malondialdehyde formation), except in the CF group, where the hexanal levels were low despite high TBARS values. However, other oxidation-derived compounds, such as isopropyl hydroperoxide (classified as “others”), which is derived from lipid oxidation processes in cooked meat, presented the highest levels in CF burgers. Hydroperoxides are intermediate compounds formed from oxidation reactions from unsaturated fatty acids in meat. These hydroperoxides further degrade into secondary volatiles like aldehydes, ketones, and alcohols [69]. These early-stage oxidation intermediates, particularly hydroperoxides, are critical in initiating thermal degradation cascades that may lead to PAH formation during high-temperature cooking [70].

Hydrocarbon levels varied with RGP addition. Hydrocarbons are also products of lipid oxidation. The production of alkanes is due to the incomplete combustion of meat and fat during grilling [67]. Our results suggest that moderate levels of RGP may increase the formation of certain volatile compounds due to interactions with meat matrices, while higher RGP concentrations could exert stronger antioxidant effects, effectively inhibiting their formation. The presence of linear and aromatic hydrocarbons in grilled meat products is closely associated with lipid oxidation processes. Lipid oxidation, a primary mechanism of deterioration in cooked and processed meats, results in the formation of volatile compounds that impact both flavor and quality. Specifically, compounds like hexane and cyclohexane derivatives are secondary products of lipid peroxidation, which occur when unsaturated fatty acids in lipids react with oxygen, generating various aldehydes, ketones, and hydrocarbons [52]. Studies have shown that the reduction or absence of these hydrocarbons in meat products often indicates an effective inhibition of lipid oxidation, commonly achieved through antioxidants that neutralize free radicals involved in the oxidation chain reaction [71]. In the same way, ketones, such as 2-Pentanone, 3-methyl- and 2-Hexanone, 4-methyl-, showed the highest levels in the control and low-RGP burgers, with significant reductions at the 3% RGP inclusion. In this context, natural antioxidants, such as those found in red grape pomace, could selectively inhibit the oxidative pathways responsible for these volatile compounds, effectively slowing down lipid degradation and maintaining product quality. Given that both hydrocarbons and ketones are considered relevant intermediates in PAH-generating pathways during grilling, their reduction in RGP-enriched burgers suggests a potential suppression of PAH precursors through polyphenol-mediated oxidative control [52].

Alcohols like 1,3-Butanediol increased at lower RGP levels but stabilized or decreased at higher levels. The formation of 1,3-Butanediol in cooked meat products is primarily linked to carbohydrate and amino acid degradation pathways, rather than lipid oxidation. This compound can increase at lower levels of red grape pomace (RGP, e.g., 0.5%), likely due to additional catalytic reactions from pomace components. However, at higher RGP levels, antioxidant activity might inhibit these reactions, limiting 1,3-Butanediol formation. These results are consistent with other studies indicating that some alcohols originate from non-lipid oxidation routes during thermal processing, particularly under intense heat [72,73].

Sulfur and nitrogen compounds in cooked meat are largely formed through Maillard reaction [74,75]. Sulfur compounds, key odor-active elements, contribute to savory profiles, while nitrogen compounds lend roasted and nutty flavors [66,76]. In RGP-enriched burgers, sulfur compounds like 1-Propene, 3,3′-thiobis-, reach higher concentrations with the 0.5% RGP, while others, such as Dihydro-4,4-dimethyl-2(3H)-furanone, remain stable or increase slightly at a higher concentration (1% and 3% RGP). This may be due to RGP’s antioxidant action, selectively inhibiting lipid oxidation while allowing Maillard sulfur reactions to persist [73]. Conversely, nitrogen compounds showed limited variation, with certain pyrazines absent at high RGP levels. This suggests that antioxidants in the RPG may suppress the heat-induced reactions responsible for nitrogen volatiles, as lipid oxidation-derived compounds promote the development of Maillard compounds during meat cooking [72]. This effect aligns with findings that antioxidants can shift volatile profiles by modulating oxidative reactions, which can indirectly impact nitrogen-based compounds, as observed in studies of antioxidant-rich herbs in meat [77]. Notably, some nitrogenous intermediates and Maillard-derived heterocyclic amines have been reported to act as precursors for high-molecular-weight PAHs in grilled meats [52]. Therefore, the observed suppression of pyrazines at high RGP levels may point to a reduced availability of reactive nitrogen species capable of participating in PAH formation pathways.

Terpene levels increased slightly with the RGP or metabisulfite addition, likely due to the antioxidant protection of spice-derived volatiles. This is consistent with findings that terpenes often enhance flavor profiles when exposed to moderate antioxidant levels, while higher RGP levels may reduce volatile concentrations due to their antioxidant activity [77]. The effect of the natural antioxidants of pomace was equivalent to the effect of the metabisulfite in the CF burgers, preserving terpenes from the spices added to the burgers.

Taken together, the observed reductions in key volatile groups (including hexanal, isopropyl hydroperoxide, branched hydrocarbons, and ketones) at higher RGP concentrations point to a lower presence of reactive intermediates associated with PAH formation. As highlighted by Du et al. [63], lipid-derived volatiles, such as hydroperoxides and short-chain aldehydes, are critical precursors in PAH synthesis during grilling. In line with this, Beriain et al. [64] demonstrated that reducing the availability of these volatile intermediates, whether through alternative cooking fuels or oxidative inhibition, significantly lowered the PAH levels in grilled meats. Therefore, the modulation of these volatiles in our RGP-formulated burgers not only reflects oxidative control but may also represent a chemical mechanism contributing to the observed low PAH content.

## 5. Conclusions

Integrating red grape pomace (RGP) into barbecued pork burgers improved the fatty acid profile and enhanced the fat content without affecting the moisture or protein levels. The RGP addition significantly reduced the lipid oxidation compared to both control groups, outperforming metabisulfite, and did so without increasing harmful PAH levels.

RGP selectively modified the volatile compound profile, decreasing hydrocarbon concentrations at higher inclusion levels, while sulfur compounds, terpenes, alcohols, and ketones showed variable responses. Although the RGP reduced lipid-derived volatiles, higher concentrations may alter the burgers’ original flavor.

These findings support the hypothesis that the whole RGP can mitigate oxidative damage and contribute to PAH management under grilling conditions, providing both safety and sensory benefits. One limitation of this study is that only a preliminary sensory screening was conducted to determine the maximum acceptable RGP level, without a full analysis of its impact on detailed sensory attributes or consumer perception. Future research should confirm its viability as a clean-label alternative to metabisulfites in burger manufacturing while also addressing sensory implications more comprehensively.

## Figures and Tables

**Table 1:** Physical–chemical composition, fatty acid profile, antioxidant activity, and phenolic compounds of the valorized red grape pomace (RGP).

Valorized Red Grape Pomace (RGP)	
pH	3.9 ± 0.0
Aw	0.980 ± 0.003
Proximate composition (% WB)	
Moisture	57.04 ± 0.6
Protein	4.3 ± 0.6
Fat	3.9 ± 0.2
Fiber	25.4 ± 1.7
Fatty acids profile (%)	
C12:0	0.1 ± 0.0
C14:0	0.1 ± 0.0
C16:0	9.5 ± 0.0
C16:1	0.4 ± 0.0
C17:0	0.1 ± 0.0
C17:1	0.0 ± 0.0
C18:0	4.6 ± 0.0
C18:1	19.4 ± 0.1
C18:2	64.5 ± 0.0
C18:3	1.0 ± 0.0
C20:0	0.2 ± 0.0
C20:1	0.1 ± 0.0
Antioxidant activity (mM Trolox/mL)	45.3 ± 3.5
Phenolic compounds (mg GAE 100g^−1^)	486.0 ± 24.5

Each value is the mean ± SD.

**Table 2:** Proximate composition (g 100g^−1^) and fatty acid profile (%) of barbecued pork burgers.

	NC			CF			0.5% RGP			1% RGP			3% RGP			*p*-Value
Moisture	57.8	±	0.8	55.8	±	1.6	57.5	±	2.5	55.0	±	2.0	56.0	±	2.5	0.273
Protein	29.7	±	0.5	31.2	±	2.1	29.8	±	1.1	32.2	±	2.4	31.2	±	2.5	0.346
Fat	5.7 ^b^	±	0.8	5.5 ^b^	±	1.0	6.1 ^ab^	±	0.2	6.9 ^a^	±	0.7	6.8 ^a^	±	0.3	0.005
Fatty acids profile (%)																
C12:0	0.08	±	0.00	0.09	±	0.00	0.09	±	0.00	0.09	±	0.00	0.09	±	0.00	0.129
C14:0	1.29	±	0.02	1.31	±	0.01	1.30	±	0.02	1.30	±	0.02	1.29	±	0.02	0.358
C16:0	23.11 ^a^	±	0.07	23.07 ^a^	±	0.05	23.16 ^a^	±	0.03	23.11 ^a^	±	0.03	22.98 ^b^	±	0.08	0.001
C16:1	3.82 ^ab^	±	0.15	3.72 ^ab^	±	0.02	3.66 ^b^	±	0.03	3.78 ^ab^	±	0.13	3.84 ^a^	±	0.02	0.045
C17:0	0.29	±	0.05	0.25	±	0.01	0.26	±	0.00	0.28	±	0.02	0.30	±	0.01	0.081
C17:1	0.26	±	0.03	0.23	±	0.00	0.24	±	0.01	0.24	±	0.00	0.26	±	0.01	0.049
C18:0	10.55	±	0.11	10.57	±	0.05	10.62	±	0.13	10.63	±	0.06	10.54	±	0.05	0.360
C18:1	44.61 ^a^	±	0.26	44.59 ^a^	±	0.20	44.42 ^a^	±	0.19	44.25 ^a^	±	0.22	43.83 ^b^	±	0.09	0.000
C18:2	14.65 ^b^	±	0.16	14.81 ^b^	±	0.27	14.91 ^b^	±	0.17	14.99 ^b^	±	0.17	15.56 ^a^	±	0.13	0.000
C18:3	0.61	±	0.01	0.61	±	0.02	0.62	±	0.01	0.61	±	0.01	0.62	±	0.01	0.459
C20:0	0.10 ^a^	±	0.02	0.10 ^a^	±	0.01	0.09 ^ab^	±	0.01	0.09 ^ab^	±	0.02	0.07 ^b^	±	0.01	0.028
C20:1	0.64 ^a^	±	0.01	0.63 ^ab^	±	0.01	0.63 ^ab^	±	0.01	0.63 ^ab^	±	0.01	0.62 ^b^	±	0.01	0.022

Each value is the mean ± SD. On the same row, means with different letters differ significantly (*p* < 0.05). NC: negative control; CF: commercial formulation; RGP: red grape pomace.

**Table 3:** Instrumental color and lipid oxidation (mg MDA kg^−1^) of grilled red pomace burgers.

	NC			CF			0.5% RGP			1% RGP			3% RGP			*p*-Value
CIE L*	49.8 ^a^	±	1.4	51.0 ^a^	±	1.2	48.4 ^ab^	±	1.9	46.0 ^b^	±	1.5	46.4 ^b^	±	0.5	*0.000*
CIE a*	7.3 ^b^	±	0.4	7.9 ^a^	±	0.1	6.1 ^c^	±	0.2	5.5 ^d^	±	0.2	4.2 ^e^	±	0.4	*0.000*
CIE b*	14.0 ^a^	±	0.7	14.3 ^a^	±	0.7	11.9 ^b^	±	1.1	10.1 ^c^	±	1.2	9.4 ^c^	±	0.6	*0.000*
Chroma	40.1 ^a^	±	1.7	43.0 ^a^	±	1.8	36.1 ^b^	±	2.5	31.5 ^c^	±	2.5	30.9 ^c^	±	1.2	*0.000*
Hue	40.7 ^a^	±	1.6	43.4 ^a^	±	1.8	37.0 ^b^	±	2.4	32.5 ^c^	±	2.5	32.0 ^c^	±	1.2	*0.000*
Lipid oxidation	0.4 ^a^	±	0.1	0.4 ^a^	±	0.2	0.1 ^b^	±	0.1	0.1 ^b^	±	0.0	0.2 ^b^	±	0.0	*0.000*

Each value is the mean ± SD. On the same row, means with different letters differ significantly (*p* < 0.05). NC: negative control; CF: commercial formulation; RGP: red grape pomace. *L*, *a*, and *b* are parameters of the CIE Lab* color space, where *L* indicates lightness, *a* represents the red-green axis, and *b* represents the yellow-blue axis.

**Table 4:** Amount of polycyclic aromatic hydrocarbons (PAHs) (µg kg^−1^) in grilled red pomace burgers.

	NC			CF			0.5% RGP			1% RGP			3% RGP			*p*-Value
Phenanthrene	0.4	±	0.1	0.5	±	0.1	0.7	±	0.4	0.9	±	0.2	0.7	±	0.2	0.173
Fluorene	0.1	±	0.0	0.1	±	0.0	0.1	±	0.1	0.1	±	0.0	0.1	±	0.0	0.605

Each value is the mean ± SD. On the same row, means with different letters differ significantly (*p* < 0.05). NC: negative control; CF: commercial formulation; RGP: red grape pomace.

**Table 5:** Volatile compounds isolated in grilled red pomace burgers (µg kg^−1^).

	NC			CF			0.5% RGP			1% RGP			3% RGP			*p*-Value	LRI
***Aldehydes***																	
Butanal, 2-methyl-	29.8	±	46.0	68.5	±	100.0	38.1	±	26.0	50.7	±	11.1	18.8	±	14.7	0.598	--
Hexanal (*)	2809.5 ^a^	±	2142.2	368.2 ^b^	±	248.0	658.7 ^b^	±	263.1	383.7 ^b^	±	163.5	506.6 ^b^	±	338.6	0.003	609.1
Heptanal (*)	1.9	±	4.3	5.9	±	13.3	1.2	±	2.6	7.1	±	7.6	1.4	±	3.2	0.583	800.3
Benzaldehyde (*)	3.8	±	8.5	9.5	±	9.6	13.8	±	2.2	12.7	±	4.6	109.5	±	215.6	0.397	966.3
2-Decenal, (Z)-	2.4 ^b^	±	5.4	17.4 ^ab^	±	16.3	25.4 ^ab^	±	6.1	34.8 ^a^	±	14.7	32.2 ^a^	±	22.5	0.015	1038.5
Nonanal (*)	105.3	±	77.0	26.9	±	42.0	20.1	±	13.4	30.5	±	6.0	66.2	±	57.6	0.050	1112.8
Decanal (*)	0.5	±	1.0	3.8	±	6.0	1.2	±	1.2	5.5	±	2.4	3.7	±	6.0	0.304	1217.1
Hexane, 3-methyl	0.0 ^b^	±	0.0	19.8 ^ab^	±	15.4	65.5 ^a^	±	51.6	31.1 ^ab^	±	21.4	10.5 ^b^	±	8.3	0.008	--
Cyclohexane, methyl-	0.0 ^b^	±	0.0	25.0 ^ab^	±	55.8	4.4 ^b^	±	9.9	70.6 ^a^	±	47.9	29.8 ^ab^	±	29.3	0.039	699.8
Hexane, 2,4-dimethyl	36.0 ^b^	±	52.9	79.8 ^b^	±	67.1	201.7 ^a^	±	122.4	66.8 ^b^	±	43.8	59.8 ^b^	±	59.3	0.018	692.0
Pentane, 2,3,4-trimethyl-	62.3	±	85.3	53.9	±	42.6	112.6	±	71.1	58.9	±	58.7	59.1	±	43.4	0.562	677.0
Pentane, 2,3,3-trimethyl-	62.3	±	139.2	84.6	±	66.6	166.1	±	104.7	117.3	±	76.0	67.1	±	53.9	0.399	672.6
Hexane, 2,3-dimethyl-	40.2	±	86.1	57.4	±	37.6	150.9	±	92.4	74.5	±	48.4	65.7	±	54.1	0.130	665.1
Heptane, 2-methyl-	203.3	±	199.7	147.4	±	109.2	533.8	±	306.3	220.5	±	135.7	282.3	±	233.0	0.066	657.4
Heptane, 3-methyl-	0.0 ^b^	±	0.0	102.1 ^b^	±	82.4	593.8 ^a^	±	349.3	154.1 ^b^	±	97.5	348.3 ^ab^	±	304.2	0.002	648.5
Cyclohexane, 1,3-dimethyl-, cis-	0.0	±	0.0	97.9	±	76.4	74.8	±	167.3	146.7	±	89.1	8.3	±	18.5	0.097	645.1
Octane, 2,6-dimethyl	50.5	±	112.9	71.0	±	58.7	152.9	±	84.1	105.4	±	70.7	64.5	±	52.2	0.281	637.2
Cyclohexane, 1,2-dimethyl-, trans-	0.0	±	0.0	27.7	±	21.2	41.2	±	39.1	39.8	±	25.1	24.7	±	20.0	0.097	618.3
3-Ethylhexane	0.0 ^b^	±	0.0	18.2 ^b^	±	15.2	72.4 ^a^	±	44.4	30.3 ^ab^	±	19.1	31.7 ^ab^	±	27.2	0.004	707.9
Cyclohexane, 1,2-dimethyl-, cis-	640.3	±	1431.7	63.1	±	122.7	16.4	±	10.9	7.4	±	4.5	3.7	±	3.5	0.466	718.9
2-Methyloctane	0.0 ^b^	±	0.0	8.6 ^ab^	±	5.9	21.9 ^a^	±	15.1	9.3 ^ab^	±	8.4	8.0 ^ab^	±	7.8	0.016	766.2
2,5-Dimethylheptane	1.2 ^b^	±	2.7	13.6 ^ab^	±	10.4	32.3 ^a^	±	19.9	18.9 ^ab^	±	12.7	12.1 ^ab^	±	10.8	0.015	772.9
Octane, 2,2-dimethyl	0.0 ^b^	±	0.0	12.8 ^ab^	±	11.1	28.0 ^a^	±	17.0	18.4 ^ab^	±	13.4	15.1 ^ab^	±	14.5	0.035	778.8
1-Octene, 6-methyl-	0.0 ^b^	±	0.0	8.5 ^a^	±	2.9	11.2 ^a^	±	7.2	6.5 ^ab^	±	3.8	0.0 ^b^	±	0.0	0.000	788.8
Octane, 3,3-dimethyl-	0.0 ^b^	±	0.0	7.3 ^ab^	±	5.6	14.7 ^a^	±	8.7	9.6 ^ab^	±	6.1	6.3 ^ab^	±	6.0	0.016	795.2
1,3,5-Cycloheptatriene, 3,7,7-trimethyl-	2.3	±	5.2	17.1	±	14.2	15.5	±	1.8	10.5	±	9.8	15.3	±	15.7	0.218	973.4
2,2,4,6,6-Pentamethylheptane	1572.1 ^ab^	±	652.4	361.3 ^b^	±	413.5	1170.2 ^ab^	±	514.5	324.3 ^b^	±	66.8	1933.9 ^a^	±	1257.0	0.005	992.9
Dodecane	1.4 ^b^	±	3.1	5.2 ^ab^	±	8.7	1.0 ^b^	±	1.1	11.9 ^a^	±	3.1	13.5 ^a^	±	8.9	0.007	1210.2
***Alcohols***																	
Ethanol (*)	0.0	±	0.0	0.0	±	0.0	0.0	±	0.0	150.8	±	94.9	174.3	±	389.7	0.325	--
1,3-Butanediol	0.0 ^b^	±	0.0	0.0 ^b^	±	0.0	29.5 ^a^	±	4.7	14.3 ^b^	±	19.9	0.0 ^b^	±	0.0	0.000	--
1-Butanol, 3-methyl	2.7	±	5.9	12.7	±	28.3	23.4	±	52.3	28.5	±	63.7	542.0	±	716.7	0.063	687.6
3-Pentanol, 2,4-dimethyl	0.0 ^b^	±	0.0	51.4 ^ab^	±	38.5	196.0 ^a^	±	119.2	18.3 ^ab^	±	40.8	130.8 ^ab^	±	180.6	0.029	686.2
2,3-Butanediol	74.2	±	150.4	41.5	±	20.7	87.6	±	42.2	52.4	±	33.6	91.7	±	87.9	0.836	626.2
2-Isopropyl-5-methyl-1-hexanol	0.0 ^b^	±	0.0	15.5 ^ab^	±	12.0	35.1 ^a^	±	22.0	24.4 ^ab^	±	15.3	13.6 ^ab^	±	11.6	0.012	784.6
1-Decanol, 2-methyl-	0.0 ^c^	±	0.0	6.6 ^ab^	±	6.1	11.8 ^a^	±	2.0	8.1 ^a^	±	4.8	6.0 ^ab^	±	4.9	0.005	798.9
2-Nonen-1-ol	0.0	±	0.0	5.6	±	5.1	9.3	±	5.9	7.7	±	6.3	5.1	±	7.8	0.146	916.4
1-Octen-3-ol	79.5 ^a^	±	91.0	0.0 ^b^	±	0.0	0.0 ^b^	±	0.0	0.0 ^b^	±	0.0	0.0 ^b^	±	0.0	0.018	980.5
***Sulfur compounds***																	
Allyl methyl sulfide	138.9	±	236.6	385.6	±	175.2	333.4	±	220.9	348.4	±	105.8	298.4	±	235.2	0.368	--
1-Propene, 3,3′-thiobis-	1.0 ^b^	±	2.2	27.9 ^ab^	±	10.6	52.6 ^a^	±	30.7	31.5 ^ab^	±	21.7	22.9 ^ab^	±	15.4	0.007	756.0
Disulfide, methyl 2-propenyl	0.0	±	0.0	4.5	±	6.2	7.1	±	6.6	4.8	±	6.7	7.8	±	7.6	0.310	800.6
Tetrahydrofuran, 2,2,4,4-tetramethyl-	2.5 ^b^	±	5.5	10.9 ^b^	±	24.3	5.4 ^b^	±	12.1	47.8 ^a^	±	20.6	35.4 ^ab^	±	22.1	0.002	970.7
Dihydro-4,4-dimethyl-2(3H)-furanone	0.0 ^b^	±	0.0	1.3 ^b^	±	2.9	0.6 ^b^	±	1.4	7.6 ^a^	±	3.0	8.7 ^a^	±	6.5	0.001	988.9
Diallyl disulfide	2.6	±	5.9	7.1	±	5.3	7.6	±	4.0	6.9	±	3.1	6.3	±	5.0	0.486	1087.2
***Nitrogen compounds***																	
2,6-Dimethylpyrazine	4.0	±	8.9	5.1	±	7.1	0.0	±	0.0	0.0	±	0.0	0.0	±	0.0	0.332	913.0
Pyrazine, 2-ethyl-6-methyl-	1.4	±	3.1	4.4	±	7.9	1.0	±	2.2	1.0	±	2.1	0.0	±	0.0	0.506	1001.5
***Terpenes***																	
3-Thujene	1.7 ^b^	±	3.8	17.0 ^a^	±	5.8	18.2 ^a^	±	6.3	14.2 ^a^	±	5.4	12.9 ^a^	±	9.3	0.005	928.4
1R-α-Pinene (*)	1341.7	±	760.5	581.7	±	230.8	519.8	±	161.5	411.7	±	156.0	1036.8	±	1332.1	0.216	934.0
Camphene	57.1	±	52.0	32.4	±	13.9	30.4	±	9.5	26.3	±	9.1	58.1	±	70.4	0.573	949.1
L-(-)-β-Pinene (*)	1806.5	±	1175.5	472.6	±	308.1	555.6	±	346.4	91.8	±	205.3	1292.8	±	2162.2	0.151	978.1
β-Myrcene	12.9 ^b^	±	28.9	143.9 ^a^	±	60.2	145.7 ^a^	±	43.7	154.0 ^a^	±	67.6	121.7 ^a^	±	88.5	0.009	995.9
α-Phellandrene	1014.1	±	591.0	242.2	±	104.6	241.1	±	75.2	233.6	±	92.3	812.8	±	1304.8	0.189	1006.5
α-terpinene (*)	4792.9	±	4427.7	1285.4	±	841.7	1587.9	±	987.0	0.0	±	0.0	4282.0	±	9574.9	0.463	1013.9
2-Carene	10.3	±	19.2	16.6	±	6.4	19.7	±	5.8	17.5	±	7.6	16.7	±	12.5	0.759	1021.1
β-Cymene	124.9	±	274.9	12.5	±	8.0	17.0	±	10.1	0.0	±	0.0	0.0	±	0.0	0.466	1027.7
o-Cymene	498.8	±	476.0	205.1	±	84.2	197.1	±	63.2	189.1	±	88.4	514.9	±	728.8	0.467	1030.0
D-Limonene (*)	4330.0	±	2638.9	1022.1	±	416.3	1060.9	±	312.8	892.9	±	393.2	3307.5	±	5124.8	0.153	1034.6
3-Carene (*)	1.0 ^b^	±	2.2	8.2 ^ab^	±	4.4	12.1 ^a^	±	3.6	8.7 ^ab^	±	3.0	9.5 ^a^	±	7.2	0.010	1066.3
p-Mentha-1,4(8)-diene	275.6	±	301.6	40.8	±	15.8	46.9	±	14.2	37.6	±	17.0	122.9	±	182.9	0.123	1093.0
β-Linalool (*)	1.6	±	3.6	8.1	±	5.6	6.7	±	1.1	7.5	±	3.1	4.5	±	3.3	0.065	1107.9
δ-Elemene	13.0	±	17.1	6.3	±	2.3	6.6	±	2.3	3.9	±	2.0	211.7	±	424.7	0.359	1351.8
Copaene	0.7 ^b^	±	1.6	8.5 ^a^	±	3.5	8.5 ^a^	±	2.5	6.8 ^ab^	±	3.5	6.5 ^ab^	±	5.2	0.013	1388.9
Caryophyllene	235.8	±	145.3	61.5	±	23.5	62.8	±	18.6	46.3	±	29.4	181.7	±	287.3	0.176	1440.7
***ketones***																	
2-Pentanone, 3-methyl-	16.6 ^b^	±	37.0	133.0 ^a^	±	67.7	74.5 ^ab^	±	30.2	81.6 ^ab^	±	31.7	27.5 ^b^	±	25.8	0.002	--
2-Hexanone, 4-methyl-	0.0 ^b^	±	0.0	9.0 ^ab^	±	7.6	4.8 ^b^	±	2.8	17.4 ^a^	±	10.5	0.0 ^b^	±	0.0	0.001	792.5
***Acids***																	
Acetic acid (*)	4.3	±	9.7	13.7	±	30.7	16.1	±	25.1	21.8	±	3.9	9.4	±	12.4	0.669	--
***Others***																	
2-Nitrohexane	8.7	±	19.5	57.0	±	56.7	65.2	±	11.5	68.8	±	11.1	407.5	±	817.6	0.444	--
Isopropyl hydroperoxide	15.9 ^b^	±	35.5	100.1 ^a^	±	47.5	0.0 ^b^	±	0.0	0.0 ^b^	±	0.0	0.0 ^b^	±	0.0	0.000	--
Hydroperoxide, hexyl	72.4	±	155.1	0.7	±	1.6	0.0	±	0.0	0.0	±	0.0	6.7	±	11.5	0.413	774.9
Oxime-, methoxy-phenyl-_	1342.2	±	1148.8	0.0	±	0.0	0.0	±	0.0	0.0	±	0.0	513.0	±	1147.1	0.033	912.7
Heptane, 3-[(1,1-dimethylethoxy)methyl]-	1.0	±	2.2	3.8	±	3.6	3.2	±	1.9	3.4	±	2.0	0.0	±	0.0	0.057	1051.9
α,4-Dimethylstyrene	0.0 ^b^	±	0.0	1.4 ^ab^	±	0.8	3.5 ^a^	±	2.3	0.0 ^b^	±	0.0	2.1 ^ab^	±	3.0	0.022	1089.8
Capric ether	0.3 ^b^	±	0.7	2.6 ^ab^	±	0.9	4.7 ^a^	±	2.8	2.1 ^ab^	±	0.5	3.8 ^a^	±	2.9	0.015	1154.8

Each value is the mean ± SD. On the same row, means with different letters differ significantly (*p* < 0.05). NC: negative control; CF: commercial formulation; RGP: red grape pomace. * The identification of the compound was carried out by the mass spectrum and LRI identical with a commercial standard compound.

## Data Availability

Dataset available on request from the authors.
